# Review of Deep Learning Based Automatic Segmentation for Lung Cancer Radiotherapy

**DOI:** 10.3389/fonc.2021.717039

**Published:** 2021-07-08

**Authors:** Xi Liu, Kai-Wen Li, Ruijie Yang, Li-Sheng Geng

**Affiliations:** ^1^ School of Physics, Beihang University, Beijing, China; ^2^ Beijing Advanced Innovation Center for Big Data-Based Precision Medicine, School of Medicine and Engineering, Key Laboratory of Big Data-Based Precision Medicine, Ministry of Industry and Information Technology, Beihang University, Beijing, China; ^3^ Department of Radiation Oncology, Peking University Third Hospital, Beijing, China; ^4^ Beijing Key Laboratory of Advanced Nuclear Materials and Physics, Beihang University, Beijing, China; ^5^ School of Physics and Microelectronics, Zhengzhou University, Zhengzhou, China

**Keywords:** lung cancer, deep learning, automatic segmentation, organs-at-risk, radiotherapy

## Abstract

Lung cancer is the leading cause of cancer-related mortality for males and females. Radiation therapy (RT) is one of the primary treatment modalities for lung cancer. While delivering the prescribed dose to tumor targets, it is essential to spare the tissues near the targets—the so-called organs-at-risk (OARs). An optimal RT planning benefits from the accurate segmentation of the gross tumor volume and surrounding OARs. Manual segmentation is a time-consuming and tedious task for radiation oncologists. Therefore, it is crucial to develop automatic image segmentation to relieve radiation oncologists of the tedious contouring work. Currently, the atlas-based automatic segmentation technique is commonly used in clinical routines. However, this technique depends heavily on the similarity between the atlas and the image segmented. With significant advances made in computer vision, deep learning as a part of artificial intelligence attracts increasing attention in medical image automatic segmentation. In this article, we reviewed deep learning based automatic segmentation techniques related to lung cancer and compared them with the atlas-based automatic segmentation technique. At present, the auto-segmentation of OARs with relatively large volume such as lung and heart etc. outperforms the organs with small volume such as esophagus. The average Dice similarity coefficient (DSC) of lung, heart and liver are over 0.9, and the best DSC of spinal cord reaches 0.9. However, the DSC of esophagus ranges between 0.71 and 0.87 with a ragged performance. In terms of the gross tumor volume, the average DSC is below 0.8. Although deep learning based automatic segmentation techniques indicate significant superiority in many aspects compared to manual segmentation, various issues still need to be solved. We discussed the potential issues in deep learning based automatic segmentation including low contrast, dataset size, consensus guidelines, and network design. Clinical limitations and future research directions of deep learning based automatic segmentation were discussed as well.

## Introduction

Cancer is becoming the leading cause of death and the most prominent obstacle to life expectancy increases in all countries. According to GLOBOCAN 2020, it is estimated that 19.3 million new cancer cases and 9.96 million cancer deaths occurred in 2020. Lung cancer, accounting for 11.4% of all new cases, is the second most common cancer. Meanwhile, it ranks first among the cancer-related mortality worldwide, accounting for 18.0% of the total cancer death (1).

In order to control the malignant tumors and improve the quality of life of cancer patients, various cancer treatment methods have gradually emerged in addition to surgical resection (2, 3), such as chemotherapy (4), radiotherapy (5–7), thermotherapy (8–10), immunotherapy (11, 12) and so on. With radiation therapy (RT) witnessing tremendous advancements in recent years, RT plays a crucial role in lung cancer treatment (6, 7, 13–15). The success of RT depends on accurate irradiating the tumor targets while sparing the organs-at-risk (OARs) and avoiding RT-related complications. Accordingly, it is vital to segment the gross tumor volume (GTV) and OARs accurately in the RT treatment planning to deliver the prescription dose to the GTV.

Manual segmentation of the GTV and OARs is a laborious and tedious process for radiation oncologists, which could result in significant delays of RT treatment and low survival rates, especially in clinics with inadequate resources. Furthermore, the quality of manual segmentation relies on the prior knowledge and experience of the radiation oncologists. Even if they segmented the GTV and OARs according to the same guidelines, inconsistencies may still exist in the segmentation for both inter- and intra-observers. On the other hand, the automatic segmentation technique has the potential to provide efficient and accurate results (16, 17). It can not only shorten the time needed to exploit the anatomy but also allow experts to devote time to optimize RT treatment planning so that the OARs could be less irradiated. In recent years, various image segmentation techniques have been proposed, resulting in more accurate and efficient image segmentation for clinical diagnosis and treatment (18–24).

Traditional automatic segmentation techniques usually segment the target depending on the shallow features of the image such as grayscale, texture, gradient, etc. In traditional automatic segmentation techniques, the common methods are Thresholding Method (25, 26), Atlas Method (27), and Region Growing Method (28) etc. Based on the target and background needed to be segmented, appropriate grayscale thresholding is selected. According to the selected thresholding, all pixels in the image to be segmented are classified into two categories, viz target and background, to perform the segmentation task. But when the grayscale difference between the image background and the target is not significant, it is difficult to segment the image accurately and efficiently (29). The Atlas Method registers the new input image to the reference image known as an atlas template, and then the labels in the atlas templates are propagated to the new input image to finalize the delineating task (30). However, the performance of the Atlas Method is heavily reliant upon the registration algorithms and the quality of the selected atlas templates (31). The Region Growing Method manually defines sub-regions in advance, then merges the adjacent pixels with similar attributes to the pre-defined region, and finally achieves the segmentation of the target region from the background (32). Nevertheless, the Region Growing Method lacks objectivity owing to the manual selection of sub-regions. Moreover, when the color feature or location information of the organs to be segmented is similar to that of other organs, the segmentation accuracy is usually not sufficiently high.

With the development of deep learning, deep learning-based models have shown superior capabilities in medical image auto-segmentation (33). Deep learning models learn feature representation independently and utilize the learned high-dimensional abstraction to finalize segmentation tasks without manual intervention (20). Recently, several studies have proposed various deep learning based automatic segmentation techniques for lung cancer (34–42). There is not yet a review of deep learning based automatic segmentation techniques for lung cancer radiotherapy. This manuscript aims to comprehensively review the deep learning based automatic segmentation techniques on lung cancer radiotherapy. The current challenges, practical issues, and future research directions of automatic segmentation are also discussed.

## Deep Learning Based Automatic Segmentation

### Basis of Deep Learning

With significant advances in computing technique and data accumulation, deep learning as a branch of artificial intelligence is attracting increasing attention in image automatic segmentation (29, 33, 43). Along with the continual increase of the model depth, deep learning can represent more complex phenomena by hierarchically extracting features of the input data *via* the hidden layers and by repeatedly training the network with the input data, such as convolutional neural networks (CNNs) (44), fully convolutional networks (FCNs) (45), and U-Net (46).

**Figure 1:**
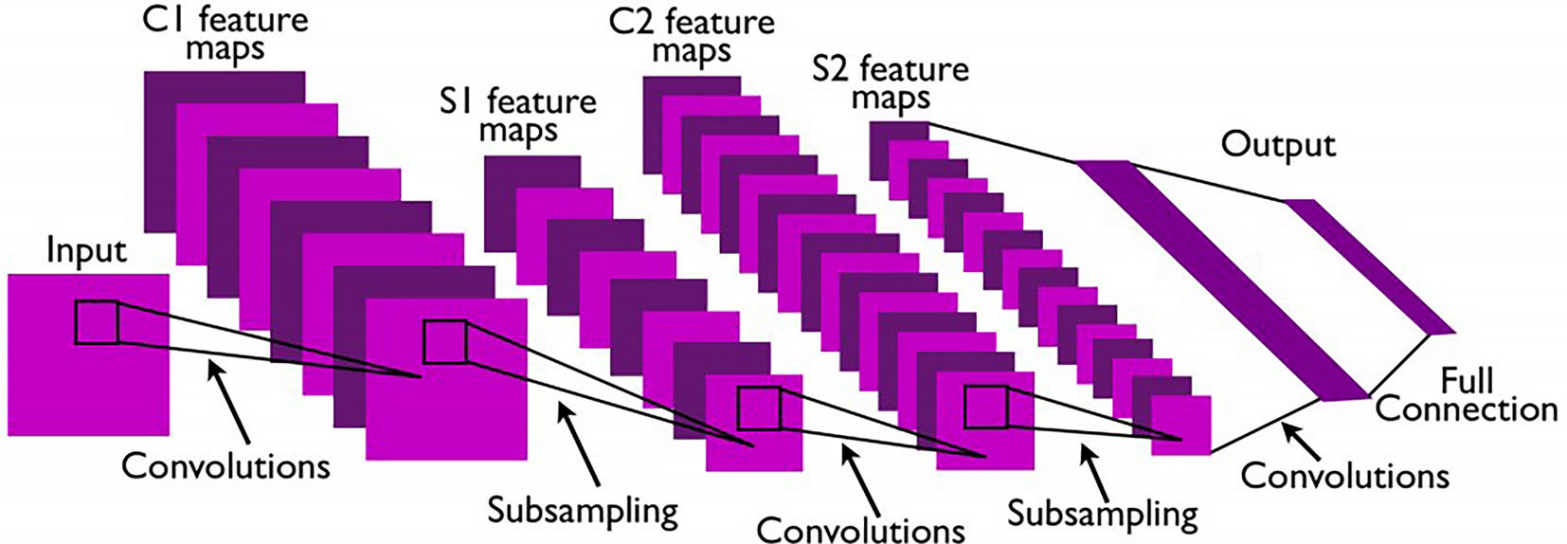
Architecture of a classic CNN (44).

As shown in **Figure 1**, CNNs (44) are generally feedforward neural networks composed of convolutional layers, pooling layers, and fully connected layers. In principle, CNNs allow to classify each individual pixel in the image, whereas the training of CNNs becomes time-consuming and computationally expensive. Although the CNN models can automatically extract image features, the pooling layers also reduce the image’s resolution while shrinking the size of the feature maps. Also, the fully connected layers have a fixed number of nodes, which limits the size of the input images.

**Figure 2:**
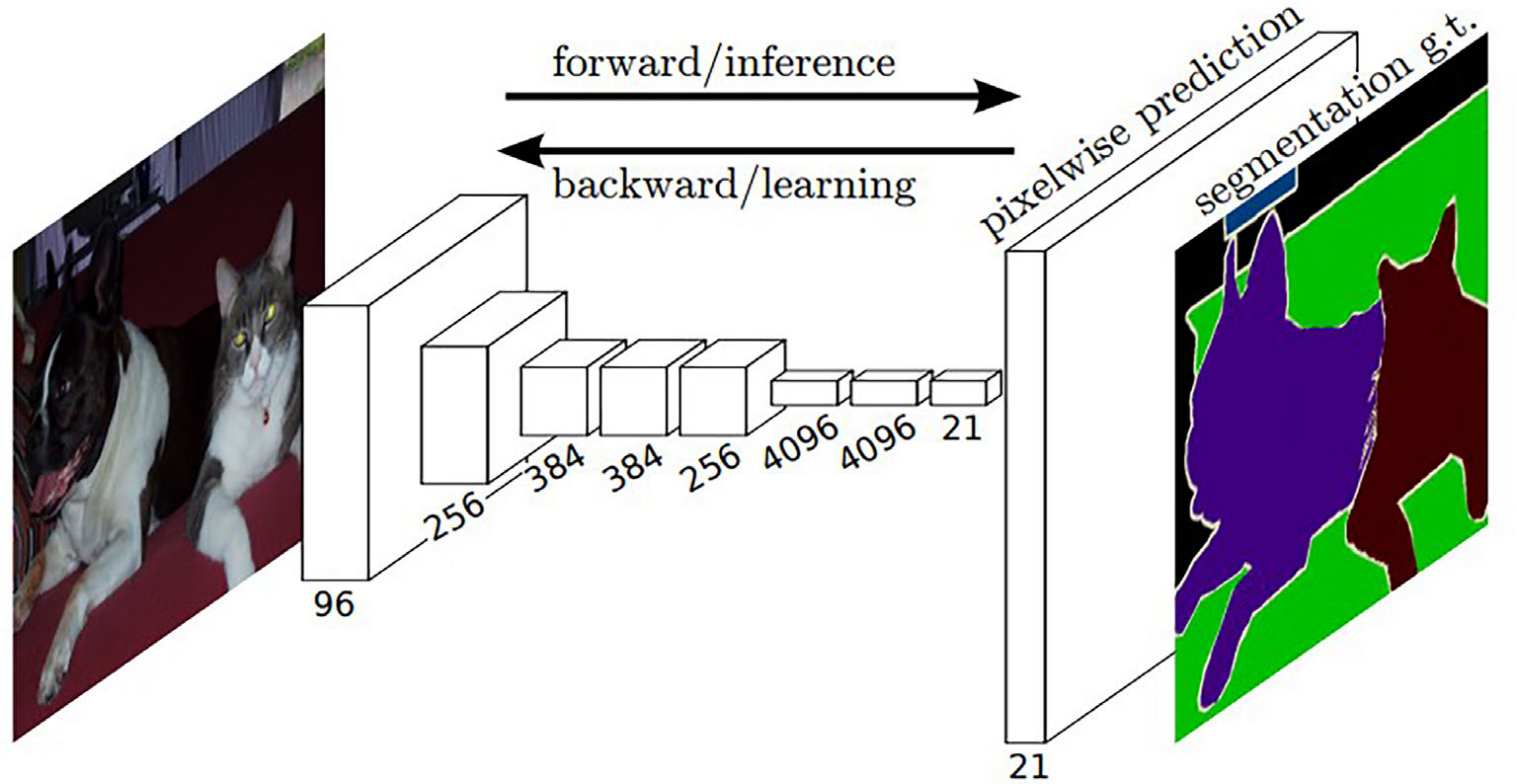
Architecture of a typical FCN (45). The white boxes represent multi-channel feature maps after the convolutional operation.

In 2015, Long et al. (45) proposed fully convolutional networks (FCNs) based on the improvement of CNNs. FCNs replace the final fully-connected layers of CNNs with the convolutional layers so that FCNs can accept input images of any size. The skip connections in FCNs improve the efficiency of image segmentation and combine the context information of the image simultaneously. **Figure 2** is a typical FCN. However, the problem is that the multiplier used in the FCNs upsampling operation is too large, resulting in the loss of segmentation accuracy and insufficient integration of context information.

**Figure 3:**
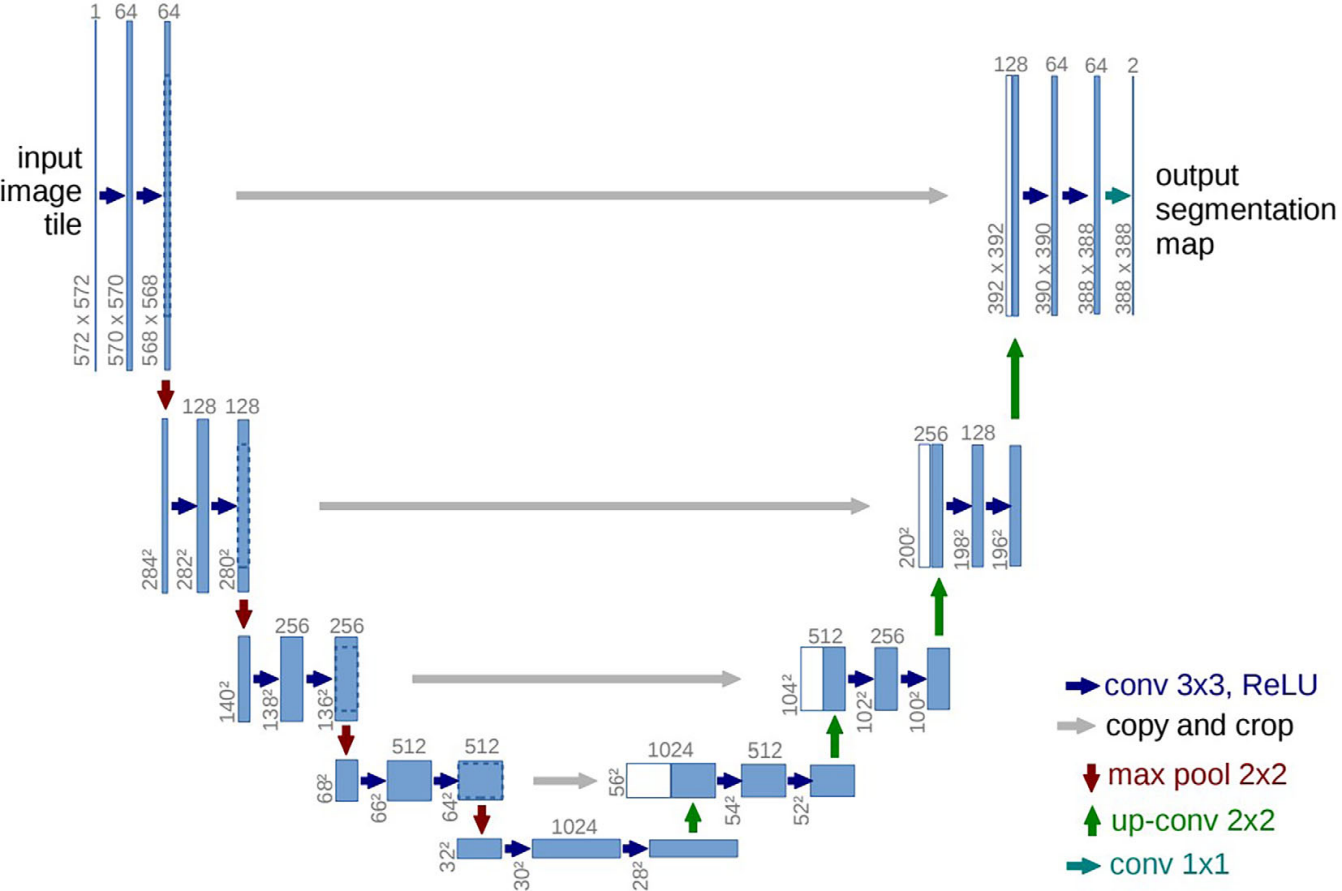
Architecture of a conventional U-Net (46). The blue boxes represent multi-channel feature maps, and the white boxes correspond to the copy of feature maps in the encoder branch. The arrows of different colors represent various operations. The number provided on the top of the box represents the number of channels, and the x-y-size of feature map is denoted at the lower left edge of the box.

To solve this problem, Ronneberger et al. (46) proposed the U-Net architecture (as illustrated in **Figure 3**), which uses the same number of convolutional layers in upsampling and downsampling. In addition, a skip connection exists between each level of the upsampling layer and the correspondingly downsampling layer, which enables the features extracted by the downsampling layer to be passed to the upsampling layer. The above-mentioned two improvements make U-Net more accurate in the aspect of pixel positioning and segmentation.

### Segmentation of OARs and GTV for Lung Cancer

The pathological characteristics of lung cancer are more complex compared with other malignant tumors. Early clinical diagnosis of lung cancer is often difficult, so that the majority of patients diagnosed with lung cancer have reached the advanced stage. The five-year survival rate of patients with advanced stage lung cancer is less than 15%, but this value can reach 40~70% if diagnosed at the early stage (47). Therefore, the early diagnosis and treatment of lung cancer is the key to improving the curative ratio. Employing deep learning in clinical practice may potentially shorten the unnecessary time and alleviate the workload of relevant staff (48). In recent years, several deep learning based automatic segmentation techniques have been proposed successively (35, 49–56). In this section, studies related to the deep learning based automatic segmentation of the OARs and GTV in lung cancer are discussed and compared. A manual searching with keywords “lung cancer, automatic segmentation, and deep learning” was carried out on three academic electronic databases viz. Web of Science, PubMed, and IEEE Xplore. Studies published from 2018~2020 are selected in this review.

#### OARs Segmentation

The precise segmentation of OARs is of vital importance to optimize the delivery of decreased dose to normal tissues, and it strongly affects the quality and outcome in lung cancer RT. Some studies have been published with regard to the segmentation of OARs in lung cancer utilizing deep learning algorithms.

In 2018, Zhao et al. (35) proposed a FCN-based network to segment lung with various diseases. In their design, they introduced a multi-instance loss to facilitate updating the most input-related convolution kernels during iterative training, and employed a conditional adversary loss to assist in correcting the lung segmentation mask. The DSC they achieved over three datasets was 0.9176, 0.9613, and 0.9793, respectively. Agnes et al. (57) trained a CNN-based network to segment lung in low-dose chest computed tomography (CT) images. They reported a mean DSC of 0.95 on LIDC-IDRI database (58). Zhu et al. (59) developed a CNN-based deep learning algorithm to automatically segment multiple thoracic OARs, including lungs, heart, spinal cord, esophagus, and liver. In their design, the network architecture was adapted from U-Net, but replacing the convolutional layer with a residual convolutional unit. In their method, the DSC of lungs, heart, spinal cord, esophagus, and liver was 0.95 ± 0.01, 0.91 ± 0.03, 0.79 ± 0.03, 0.71 ± 0.05, and 0.89 ± 0.02, respectively.

In 2019, Dong et al. (38) proposed a U-Net-GAN strategy to automatically contour left and right lungs, spinal cord, esophagus and heart. Their design adopted the architecture of generative adversarial network (GAN), employing the U-Nets as generators and the FCNs as discriminators. They achieved a DSC of 0.97 ± 0.01, 0.97 ± 0.01, 0.90 ± 0.04, 0.87 ± 0.05, and 0.75 ± 0.08 for the left lung, right lung, spinal cord, heart, and esophagus, respectively. Correspondingly, the mean surface distance (MSD) was 0.61 ± 0.73, 0.65 ± 0.53, 0.38 ± 0.27, 1.49 ± 0.85, and 1.05 ± 0.66 mm. The average sensitivity of the proposed method was 0.74 ~ 0.97, with the best for the lung and the worst for the esophagus. Additionally, they compared the performance of U-Net with and without the adversarial network. They concluded that with the assistance of the adversarial network, the segmentation accuracy was improved, and the biggest improvement was found for the spinal cord.

Later, Feng et al. (39) developed another novel segmentation model based on 3D U-Net for the automatic segmentation of five thoracic OARs, including left and right lungs, heart, esophagus and spinal cord. In their model, given that each organ has a relatively fixed position within the CT images, they firstly cropped the original 3D images into smaller patches ensuring each patch containing only one organ to be segmented. Secondly, for each organ, an individual 3D U-Net was trained to segment the organ from the cropped patches. The individual segmentation results were resampled and integrated together to generate the final segmentation results. According to their testing, the model segmented the OARs with a mean DSC of 0.893, 0.972, 0.979, 0.925, 0.726 for the spinal cord, right lung, left lung, heart and esophagus, respectively. The MSDs were as follows: (spinal cord: 0.662 ± 0.248 mm, right lung: 0.933 ± 0.574 mm, left lung: 0.586 ± 0.285 mm, heart: 2.297 ± 0.492 mm, esophagus: 2.341 ± 2.380 mm).

In the same year, Trullo et al. (60) organized a competition called SegTHOR on the theme “Automatic segmentation of Organs-at-risk in Thoracic CT images”. In the competition, various segmentation techniques based on different frameworks were proposed to automatically delineate four OARs: heart, aorta, trachea, esophagus. Among all the techniques based on CNN architecture, Harten et al. (61) obtained the best performance in terms of DSC (esophagus: 0.84, heart: 0.94, trachea: 0.91, and aorta: 0.93) and Hausdorff distance (HD) (esophagus: 3.4 mm, heart: 2.0 mm, trachea: 2.1 mm, and aorta: 2.7 mm). They combined a 2D CNN with a 3D CNN to segment the OARs. The 2D CNN containing dilated convolutions performed multi-class segmentation while the 3D CNN containing residual blocks performed multi-label segmentation, promoting additional diversity in the networks.

Among all the techniques based on U-Net architecture, He et al. (62) obtained the highest value of DSC and lowest HD for esophagus (DSC: 0.8594; HD: 0.2743), heart (DSC: 0.9500; HD: 0.1383) and aorta (DSC: 0.9484; HD: 0.1129). They proposed a uniform U-like encoder-decoder architecture abstracted from the U-Net and trained it under the multi-task learning schema. Commonly used network architecture such as ResNet and DenseNet could be involved in the encoder part by eliminating their linearly connected layers, and the encoder could adopt the transfer learning under this design. It is the transfer learning that shortens the training time and boots the performance of the network. With regard to the trachea, Vesal et al. (63) achieved a better performance than He et al. with a DSC of 0.926 and an HD of 0.193 mm. They modified the 2D U-Net mainly in two aspects. Firstly, dilated convolutions were introduced to expand the receptive fields so that both local and global information was used efficiently without increasing the network complexity. Additionally, to better incorporate multi-scale image features, the convolution layers were replaced with the residual convolution layers in the encoder branch.

Among all the techniques based on V-Net architecture, the best result for the esophagus (DSC: 0.8651; HD: 0.2590 mm), heart (DSC: 0.9536; HD: 0.1272 mm), trachea (DSC: 0.9276; HD: 0.1453 mm) and aorta (DSC: 0.9464; HD: 0.1209 mm) was obtained by Han et al. (40) who was the winner of the SegTHOR competition. Based on V-Net, they proposed a novel framework called multi-resolution VB-Net. The network consists of two parts: (a) the contraction path on the left side to extract high-level contextual information of the input data employing convolution and downsampling; (b) the expansion path on the right side to integrate high-level contextual information with detailed local information *via* skip connections to improve the accuracy of the outputs. They utilized the bottleneck structure to replace the conventional convolutional layers inside the upsampling and downsampling processes. Furthermore, to reduce the GPU memory and computation cost, they adopted a multi-resolution strategy. They trained two VB-Nets separately, one in the coarse resolution to roughly get the location of the ROI for each organ and the other in the fine resolution to accurately delineate the OAR boundaries from the detected ROI.

In 2020, Zhang et al. (64) established a CNN network based on ResNet-101 for automatically segmenting the OARs, including lungs, esophagus, heart, liver, and spinal cord. They reported a mean DSC of 0.948, 0.943, 0.821, 0.893, 0.937 and 0.732 for the left lung, right lung, spinal cord, heart, liver, and esophagus, respectively. Correspondingly, the MSD was 1.10 ± 0.15, 2.23 ± 2.33, 0.87 ± 0.21, 1.65 ± 0.48, 2.03 ± 1.49 and 1.38 ± 0.44 mm.

Hu et al. (65) used Mask R-CNN architecture combined with supervised (Bayes, Support Vectors Machine) and unsupervised (K-means and Gaussian Mixture Models) machine learning methods to segment lungs on CT images automatically. Mask R-CNN consists of two stages: (a) a Region Proposal Network to generate candidate object bounding boxes and predict the classes of objects; (b) while predicting the class and box offset, an FCN to generate a binary mask for each detected object. They concluded that the method combining Mask R-CNN with the K-means kernel generated the best results for lung segmentation with a DSC of 97.33 ± 3.24% and a sensitivity of 96.58 ± 8.58%.

Apart from various CNN models, GAN models have also been utilized for image segmentation. Tan et al. (66) proposed a new schema called LGAN for lung segmentation based on the architecture of GAN. Meanwhile, a novel loss function based on the Earth Mover distance was used in their schema. For this schema, a generative network (generator) is constructed to produce the lung mask, and a discriminative network (discriminator) is constructed to differentiate the generated synthetic maps from the ground truth. The generator and discriminator are trained sequentially and iteratively in a competing way to boost the performance of the other, which assists the generator to generate lung segmentation results that cannot be differentiated from the ground truth. After exploring various discriminator designs for lung segmentation, they achieved the best performance by designing the discriminative network as a regression network. This proposal had an Intersection over Union (IOU) of 0.923 and an HD of 3.380 mm on the LIDC-IDRI dataset in which the patients were selected from the public database founded by the Lung Image Database Consortium and Image Database Resource Initiative. They also evaluated the proposal on another private dataset, achieving an IOU of 0.938 and an HD of 2.812 mm. Considering both using the LIDC-IDRI dataset, a comparison between LGAN and U-Net (46) had been conducted. According to the results of the paper, the DSC of LGAN and U-Net in [mean, median] form were [0.970 ± 0.59, 0.9845] *vs.* [0.985 ± 0.03, 0.9864]. A better DSC indicates that LGAN outperforms commonly used U-Net.

Pawar et al. (67) developed a deep learning algorithm to effectively segment lung, which is denoted as LungSeg-Net. LungSeg-Net is similar to GAN, including two networks, viz. generator and discriminator. The generator is composed of three major components: (a) encoder block to extract feature maps; (b) multi-scale dense feature extraction module to extract multi-scale features from the set of encoded feature maps; (c) decoder block to generate the output lung segmentation map from the multi-scale features. They compared the performance of the proposed LungSeg-Net with the existing state-of-the-art CNNs viz. U-Net (46), ResNet (68), VGG16 (69). The DSC of lung achieved with the proposed network ranges between 0.9140 and 0.9899 on average. They concluded that the LungSeg-Net showed considerable performance improvement compared to other CNNs in the lung segmentation with different interstitial lung disease patterns.

At present, OARs segmentation is currently limited to delineate the whole organ in the majority of studies related to automatic segmentation. Yet, there is evidence suggesting that dose to sensitive cardiac substructures may give rise to cardiac toxicities (70–72) involving cardiomyopathy, coronary artery disease etc. (73). Especially, coronary artery calcification onset has been more relevant to the maximum dose to the left anterior descending artery, compared to the mean heart dose (74). However, because of the limited ability to contour these sensitive cardiac substructures, these dose thresholds are unavailable. Recently, Morris et al. (75) explored the accurate segmentation of twelve cardiac substructures, including chambers, great vessels, coronary arteries, etc. They proposed a 3D U-Net combined with a fully connected conditional random field to automatically segment cardiac substructures. Eventually, they obtained acceptable segmentations for chambers (DSC: 0.88 ± 0.03), great vessels (DSC: 0.85 ± 0.03), and pulmonary veins (DSC: 0.77 ± 0.04), compared to inferior performance on coronary arteries (DSC: 0.50 ± 0.14). In terms of further refinement of coronary artery segmentation, the author stated that utilizing conditional random fields as RNNs may be worth of studying.

It’s also worth noting that most works focused on segmenting OARs using deep learning based algorithms in single energy CT images. On the other hand, dual energy CT which enables to acquire two different CT images concurrently (76) could supply higher contrast and more information about differences between tissues, compared with single energy CT. Therefore, using dual energy CT as input of deep learning network may help achieve more accurate segmentation (77, 78). Chen et al. (78) designed four 3D FCNs on basis of U-Net and ResNet for automatically segmenting the OARs using dual energy CT images. The four networks merged the extra information into the network in different ways: (a) linearly combining dual energy images into one mixed image as the input; (b) using the dual energy images as two channels of the same input; (c) extracting features of the low energy image and the high energy image separately and fusing them at the bottom of the U-Net; (d) handling the low energy image and the high energy image separately and fusing the prediction results into one finally. According to their test results, the best mean DSC were 97.5 ± 0.64%, 97.6 ± 1.61%, and 96.2 ± 1.64%, for the left lung, right lung, and liver, separately.

**Table 1** is a brief summary of the aforementioned works. **Tables 2**–**6** shows the comparison of selected works on the segmentation of different OARs.

**Table 1:** Selected works on deep learning-based automated segmentation of OARs for lung cancer.

Reference	Year	Networks	OARs	Research Highlight
Zhao et al. (35)	2018	FCN	lung	introducing a multi-instance loss and a conditional adversary loss to facilitate more correct segmentation
Agnes et al. (57)	2018	U-Net	lung	exploring the performance of different convolutional network configurations, and comparing proposed model with other methods such as the thresholding method etc.
Zhu et al. (59)	2018	U-Net	lung, heart, esophagus, spinal cord, and liver	replacing the common convolutional layers with residual convolution units
Dong et al. (38)	2019	U-net-GAN	left lung, right lung, heart, esophagus, and spinal cord	proposing a 2.5D patch-based GAN to delineate the left lung, right lung, and heart, two 3D patch-based GAN to delineate esophagus and spinal cord separately
Feng et al. (39)	2019	U-Net	left lung, right lung, heart, esophagus, and spinal cord	developing two 3D U-Nets, one for localizing each OAR and the other for individually segmenting each OAR
Harten et al. (61)	2019	CNN	heart, aorta, trachea, esophagus	combining a 2D CNN which utilizes dilated convolutions with a 3D CNN which employs residual blocks
He et al. (62)	2019	adapted U-Net	heart, aorta, trachea, esophagus	proposing a U-like architecture in which the encoder could set diverse network framework, and training it under the multi-task learning schema
Vesal et al. (63)	2019	U-Net	heart, aorta, trachea, esophagus	employing dilated convolutions in the bottleneck of a 2D U-Net styled network and residual connections in the encoder branch
Han et al. (40)	2019	V-Net	heart, aorta, trachea, esophagus	proposing a multi-resolution VB-Net architecture by replacing the convolutional layers inside the V-Net with a bottleneck framework
Zhang et al. (64)	2020	CNN	left lung, right lung, heart, esophagus spinal cord, and liver	establishing a dilated CNN structure based on ResNet-101, and comparing its performance with atlas-based and manual segmentation
Hu et al. (65)	2020	Mask R-CNN	lung	using a Mask R-CNN combined with supervised and unsupervised machine learning methods to segment the lung
Tan et al. (66)	2020	GAN	lung	proposing a GAN-based architecture combing a novel loss function based on the Earth Mover distance for lung segmentation
Pawar et al. (67)	2020	c-GAN	lung	introducing a c-GAN structure that used multi-scale dense feature extraction blocks for the lung segmentation with different interstitial lung disease patterns
Morris et al. (75)	2020	U-Net	cardiac substructures	proposing a 3D U-Net combined with fully connected conditional random fields to segment twelve cardiac substructures
Chen et al. (78)	2020	FCN	left lung, right lung, liver, spleen, left kidney, and right kidneys	proposing a 3D FCN based on the U-Net and ResNet to segment OARs in dual energy CT images

FCN, fully convolutional network; GAN, generative adversarial network; OAR, organ-at-risk; 3D, three dimensional; 2D, two dimensional; CNN, convolutional neural network; R-CNN, Region- convolutional neural network; c-GAN, conditional generative adversarial network.

**Table 2:** Comparison of selected works on segmentation of lung.

Reference	Year	Networks	Evaluation Metrics						
			DSC	IOU	HD (mm)	95%HD (mm)	MSD (mm)	Sensitivity	Specificity
Zhao et al. (35)	2018	FCN	LIDC: 0.92	–	–	–	–	–	–
			CLEF: 0.96						
			HUG: 0.98						
Agnes et al. (57)	2018	U-Net	0.95 ± 0.03	–	–	–	–	0.95 ± 0.03	0.99 ± 0.01
Zhu et al. (59)	2018	adapted U-Net	0.95 ± 0.01	–	–	7.96 ± 2.57	1.93 ± 0.51	–	–
Dong et al. (38)	2019	U-net-GAN	Left: 0.97 ± 0.01	–	–	2.07 ± 1.93	0.61 ± 0.73	0.97 ± 0.02	0.9989 ± 0.0010
			Right: 0.97 ± 0.01			2.50 ± 3.34	0.65 ± 0.53	0.96 ± 0.02	0.9992 ± 0.0007
Feng et al. (39)	2019	3D U-Net	Left: 0.98 ± 0.01	–	–	2.10 ± 0.94	0.59 ± 0.29	–	–
			Right: 0.97 ± 0.02			3.96 ± 2.85	0.93 ± 0.57		
Zhang et al. (64)	2020	ResNet-101	Left: 0.95 ± 0.01	–	–	–	1.10 ± 0.15	–	–
			Right: 0.94 ± 0.02				2.23 ± 2.33		
Hu et al. (65)	2020	Mask R-CNN	0.97 ± 0.03	–	–	–	–	0.97 ± 0.09	0.9711 ± 0.0365
Tan et al. (66)	2020	GAN	–	0.938	2.812	–	–	–	–
Chen et al. (78)	2020	3D FCN	Left: 0.98 ± 0.01	–	–	–	–	–	–
			Right: 0.98 ± 0.02						

FCN, fully convolutional network; LIDC, Lung Image Database Consortium; CLEF, Conference and Labs of the Evaluation Forum; HUG, University Hospitals of Geneva; DSC, Dice similarity coefficient; IOU, intersection over Union; HD, Hausdorff distance; 95%HD, 95% Hausdorff distance; MSD, mean surface distance; R-CNN, Region- convolutional neural network; GAN, generative adversarial network.

**Table 3:** Comparison of selected works on segmentation of esophagus.

Reference	Year	Networks	Evaluation Metrics			
			DSC	HD (mm)	95%HD (mm)	MSD (mm)
Zhu et al. (59)	2018	adapted U-Net	0.71 ± 0.05	–	7.83 ± 2.85	2.18 ± 0.80
Dong et al. (38)	2019	U-net-GAN	0.75 ± 0.08	–	4.52 ± 3.81	1.05 ± 0.66
Feng et al. (39)	2019	3D U-Net	0.73 ± 0.09	–	8.71 ± 10.59	2.34 ± 2.38
Harten et al. (61)	2019	CNN	0.85 ± 0.05	3.4 ± 2.3	–	–
He et al. (62)	2019	U-Net with multi-task learning	0.86	0.274	–	–
Vesal et al. (63)	2019	2D U-Net	0.86	0.331	–	–
Han et al. (40)	2019	VB-Net	0.87	0.259	–	–
Zhang et al. (64)	2020	ResNet-101	0.73 ± 0.07	–	–	1.38 ± 0.44

DSC, Dice similarity coefficient; HD, Hausdorff distance; 95%HD, 95% Hausdorff distance; MSD, mean surface distance; GAN, generative adversarial network.

**Table 4:** Comparison of selected works on segmentation of spinal cord.

Reference	Year	Networks	Evaluation Metrics		
			DSC	95%HD (mm)	MSD (mm)
Zhu et al. (59)	2018	adapted U-Net	0.79 ± 0.03	4.01 ± 2.05	1.25 ± 0.23
Dong et al. (38)	2019	U-net-GAN	0.90 ± 0.04	1.19 ± 0.46	0.38 ± 0.27
Feng et al. (39)	2019	3D U-Net	0.89 ± 0.04	1.89 ± 0.63	0.66 ± 0.25
Zhang et al. (64)	2020	ResNet-101	0.82 ± 0.05	–	0.87 ± 0.21

DSC, Dice similarity coefficient; HD, Hausdorff distance; 95%HD, 95% Hausdorff distance; MSD, mean surface distance; GAN, generative adversarial network.

**Table 5:** Comparison of selected works on segmentation of heart.

Reference	Year	Networks	Evaluation Metrics			
			DSC	HD (mm)	95%HD (mm)	MSD (mm)
Zhu et al. (59)	2018	adapted U-Net	0.91 ± 0.03	–	7.98 ± 4.56	2.92 ± 1.51
Dong et al. (38)	2019	U-net-GAN	0.87 ± 0.05	–	4.58 ± 3.67	1.49 ± 0.85
Feng et al. (39)	2019	3D U-Net	0.93 ± 0.02	–	6.57 ± 1.50	2.30 ± 0.49
Harten et al. (61)	2019	CNN	0.95 ± 0.01	2.0 ± 1.1	–	–
He et al. (62)	2019	U-Net with multi-task learning	0.95	0.138	–	–
Vesal et al. (63)	2019	2D U-Net	0.94	0.226	–	–
Han et al. (40)	2019	VB-Net	0.95	0.127	–	–
Zhang et al. (64)	2020	ResNet-101	0.89 ± 0.05	–	–	1.65 ± 0.48

DSC, Dice similarity coefficient; HD, Hausdorff distance; 95%HD, 95% Hausdorff distance; MSD, mean surface distance; GAN, generative adversarial network.

**Table 6:** Selected works on segmentation of other OARs (liver, aorta, trachea).

Reference	Year	Networks	Evaluation Metrics		
			DSC	HD (mm)	MSD (mm)
Zhu et al. (59)	2018	adapted U-Net	Liver: 0.89 ± 0.02	–	3.21 ± 0.93
Zhang et al. (64)	2020	ResNet-101	Liver: 0.94 ± 0.03	–	2.03 ± 1.49
Chen et al. (78)	2020	3D FCN	Liver: 0.96 ± 0.16	–	–
Harten et al. (61)	2019	CNN	Aorta: 0.93 ± 0.01	2.7 ± 3.6	–
			Trachea: 0.91 ± 0.02	2.1 ± 1.0	–
He et al. (62)	2019	U-Net with multi-task learning	Aorta: 0.95	0.113	–
			Trachea: 0.92	0.182	–
Vesal et al. (63)	2019	2D U-Net	Aorta: 0.94	0.297	–
			Trachea: 0.93	0.193	–
Han et al. (40)	2019	VB-Net	Aorta: 0.95	0.121	–
			Trachea: 0.93	0.145	–

DSC, Dice similarity coefficient; HD, Hausdorff distance; 95%HD, 95% Hausdorff distance; MSD, mean surface distance; GAN, generative adversarial network.

#### Lung Tumor Segmentation

The usage of deep learning techniques assists radiation oncologists in segmenting lung on CT or magnetic resonance imaging (MRI) images with greater accuracy, consistency, and efficiency. Diverse network architectures are established by different authors in their published papers. Owing to the state-of-the-art performance of CNNs in challenging problems, for instance, computer vision, object detection, and image recognition, researchers gradually shift to using CNNs for the GTV segmentation and auxiliary diagnosis.

Inspired by CNN architectures, Wang et al. (36) introduced a new patient-specific adaptive convolutional neural network (A-net) for automatically contouring lung tumors seen on weekly MRI images. A-net mainly consists of three convolution blocks, three fully connected blocks, and one SoftMax layer. A dropout layer comes along with a fully connected layer in the last three levels, which was used to solve the potential over-fitting problem. 2D patches with a size of 3 cm × 3 cm were cropped as inputs to A-net within the region of interest of the weekly MRI scans. A-net utilized the previous weekly MRI images and the segmentation of the GTV to train and update the network, and the current weekly MRI images were allocated as testing data. With this method, they obtained the segmentation results of the weekly MRI with a DSC and a precision of 0.82 ± 0.10 and 0.81 ± 0.10, respectively.

Zhang et al. (79) introduced another modified ResNet to segment the GTV of non-small cell lung cancer patients on the CT images. In this method, the deep features of the input data were effectively extracted using two different residual convolutional blocks. The feature maps generated at all levels of the ResNet were merged into a single output. This modification made shallow surface features fuse with the deep semantic features to generate dense pixel outputs. Utilizing the proposed modified ResNet, the average DSC level achieved is 0.73. A comparison between modified ResNet and U-Net had been conducted on the same dataset. They concluded that modified ResNet outperforms U-Net, because U-Net has a lower DSC with a mean value of 0.64.

Given that the residual connections solely employed in ResNet do not eliminate the issue of poor localization and blurring arising from consecutive pooling operations, Pohlen et al. (80) proposed the full resolution residual neural network (FRRN) which passes features at full image resolution to each layer. In 2018, Jiang et al. (41) modified the FRRN and proposed two multiple resolution residually connected network (MRRN) architectures called incremental-MRRN and dense-MRRN to automatically segment lung tumors. When combining feature maps at multiple image resolutions and feature levels, a dense feature representation is simultaneously generated so that the performance of the MRNN on recovering the input image spatial resolution is better than other networks. The main difference between incremental-MRRN and dense-MRRN is that incremental-MRRN sequentially integrates higher spatial resolution information starting from the immediately previous residual stream, whereas dense-MRRN only residually integrates information from the immediate higher spatial resolution feature maps. In the work, the performance of different networks, including U-Net (46), SegNet (81) and FRRN (80) was compared with two MRNNs. The DSC, 95%HD, sensitivity and precision of U-Net, SegNet, FRRN, incremental-MRRN and dense-MRRN in three different datasets are shown in **Table 7**. According to their research results, it could be concluded that incremental-MRRN shows more robust performance than U-Net, SegNet, and FRRN.

**Table 7:** Comparison of different networks on segmentation of lung tumors in (41).

Networks	TCIA dataset				MSKCC dataset				LIDC dataset			
	DSC	95%HD	Sensitivity	Precision	DSC	95%HD	Sensitivity	Precision	DSC	95%HD	Sensitivity	Precision
U-Net	0.68	15.51	0.73	0.71	0.65	7.87	0.75	0.66	0.58	4.95	0.80	0.64
SegNet	0.70	15.24	0.73	0.72	0.66	7.92	0.72	0.69	0.57	4.48	0.77	0.60
FRRN	0.71	12.66	0.75	0.73	0.71	7.72	0.69	0.71	0.60	2.91	0.76	0.64
incremental-MRRN	0.74	7.94	0.80	0.73	0.74	5.85	0.82	0.72	0.68	2.60	0.85	0.67
dense-MRRN	0.73	8.10	0.79	0.73	0.73	5.94	0.80	0.72	0.67	2.72	0.82	0.70

TCIA, The Cancer Imaging Archive; MSKCC, Memorial Sloan Kettering Cancer Center; LIDC, Lung Image Database Consortium; DSC, Dice similarity coefficient; 95%HD, 95% Hausdorff distance; FRRN, full resolution residual neural network; MRRN, multiple resolution residually connected network.

In addition to single-modality segmentation, multi-modality co-segmentation have also been proposed (82, 83). Zhao et al. (82) proposed a novel scheme that utilizes both positron emission tomography (PET) and CT image information concurrently for lung tumor delineating. In their scheme, the network framework consisted of two parts namely multi-task training module and feature fusion module. The multi-task training module included two parallel sub-segmentation branches used for extracting features from PET or CT image independently. Each sub-segmentation branch was designed on the basis of the V-Net which is a 3D FCN. Afterwards, two feature maps generated by two parallel branches were fed into the feature fusion module which was comprised of cascaded convolutional operations. In the feature fusion module, high-dimensional information from PET and CT were fused, and re-extracted to generate outputs. They compared the performance of the proposed scheme with scheme utilizing PET or CT only on the same dataset. The comparison of three schemes on DSC were as follows: (PET&CT: 0.85 ± 0.08; PET only: 0.83 ± 0.10; CT only: 0.76 ± 0.07).

In terms of lung cancer, deep learning algorithms proposed in various works have outperformed the existing solutions in most scenarios. However, most recent studies predominantly focused on the segmentation of the GTV, and few studies have explored the usage of this state-of-the-art technique for clinical target volume (CTV) segmentation. Bi et al. (84) established a deep dilated residual network based on ResNet-101 to automatically delineate the CTV for non-small cell lung cancer patients receiving postoperative RT. They summarized that with the assistance of dilated residual network, moderate segmentation accuracy was obtained for the CTV with a DSC of 0.75 ± 0.06. It is more challenging to segment the CTV perhaps owing to the following reasons. The postoperative CTV cannot be easily recognized by discriminating tissue density as it was for the GTV because the CTV usually contains the high-risk nodal regions and bronchial stump. Moreover, postoperative changes, for instance blurred soft tissue boundary, ectopic target due to diverse lobectomies, and a wide variety of different patient’s lung volume, possibly increase anatomical diversity. Besides, the definition of the CTV is more complex compared with organs. Inter-observer variability resulting from different practical experiences and clinical guidelines has been considered to be a huge challenge in the automated CTV delineating. More information about the aforementioned works is summarized in **Table 8**.

**Table 8:** Selected works on deep learning-based automated segmentation of lung tumors.

Reference	Year	Network	Datasets	Input	Targets		Results	Research Highlight
Wang et al. (36)	2018	CNN	9 patients	MRI		GTV	DSC: 0.82 ± 0.10	establishing a patient-specific adaptive patch-based CNN and a population-based CNN, and comparing their performance with each other
							Precision: 0.81 ± 0.10	
Zhang et al. (79)	2020	ResNet	330 patients (training set: 300; test set: 30)	CT		GTV	DSC: 0.73 ± 0.07	proposing a modified version of ResNet to segment the GTV for NSCLC patients and comparing its performance with U-net
							JSC: 0.68 ± 0.09	
							TPR: 0.74 ± 0.07	
							FPR: 0.0012 ± 0.0014	
Zhao et al. (82)	2018	3D FCN	84 patients (training set: 48; test set:36)	PET/CT		Lung tumor	DSC: 0.85 ± 0.08	proposing a multi-modality co-segmentation network and comparing its performance with utilizing CT or PET only
Jiang et al. (41)	2019	MRRN	1210 patients from three datasets (377 from TCIA for training, 304 from MSKCC for validating, and 529 from LIDC for testing)	CT		Lung tumor	form: (TCIA, MSKCC, LIDC)	developing two multiple resolution residually connected network viz. incremental-MRRN and dense-MRRN, and comparing their performance with other commonly used networks
							DSC: (0.74, 0.75, 0.68)	
							Precision: (0.73, 0.72, 0.67)	
							Sensitivity: (0.80, 0.82, 0.85)	
							95%HD (mm): (7.94, 5.85, 2.60)	
Bi et al. (84)	2019	ResNet-101	269 patients (training set: 200; validation set: 50; test set:19)	CT		CTV	DSC: 0.75 ± 0.06	introducing a deep residual network with dilated blocks to segment the CTV for NSCLC patients, and comparing with manual delineation
							MDTA: 2.97 ± 0.91	
							CV: 0.129 ± 0.040	
							SDD: 0.47 ± 0.22	

CNN, convolutional neural network; MRI, magnetic resonance imaging; GTV, gross tumor volume; DSC, Dice similarity coefficient; RMSD, root mean surface distance; ResNet, residual network; CT, computed tomography; JSC, Jaccard similarity coefficient; TPR, true positive rate; FPR, false positive rate; NSCLC, non-small cell lung cancer; MRRN, multiple resolutions residually connected network; TCIA, The Cancer Imaging Archive; MSKCC, Memorial Sloan Kettering Cancer Center; LIDC, Lung Image Database Consortium; 95%HD, 95% Hausdorff distance; CTV, clinical target volume; MDTA, mean distance to agreement; CV, coefficient of variation; SDD, standard distance deviation.

## Discussion

### Comparison Between the Atlas-Based and Deep Learning Based Automatic Segmentation

Currently, the atlas-based automatic segmentation technique is most commonly employed in clinical practice. The atlas-based automatic segmentation utilizes a reference image as an atlas, in which the boundaries of interested organs are already precisely delineated. The reference image and the new image to be segmented are registered, and the optimal transformation parameters between the two images are obtained. Then the new test image is automatically segmented by propagating the label in the atlas segmentation onto the new test image based on the obtained transformation parameters (85–87). This method has a precise segmentation result in theory, but in practice the segmentation accuracy relies heavily on the similarity between the reference image and the image to be segmented. Additionally, the choice of the deformable image registration algorithm plays a vital role in the performance of segmentation. Due to organ morphology, variety of the individual patient, and image artifacts, accurate image registration is not always ensured. While this issue may be mitigated with a larger and more diverse atlas dataset, it is difficult to contain all potential patterns in the templates given the unpredictability of tumor shape. Besides, accurate image registration is costly in computation, and a large number of atlas templates make the computation cost soaring with the increase in the segmentation accuracy (38, 88–90).

Several studies compared the performance of automatic segmentation using atlas-based and deep learning based techniques separately to delineate the OARs in lung cancer, as shown in **Table 9**. Lustberg et al. (91) compared user adjusted contours after an atlas-based and deep learning based delineation, against manual contours. In terms of the time saved, they reported that the total median was 7.8 min and 10 min for using atlas-based and deep learning based contouring software, respectively. With regard to the esophagus, deep learning based contouring software outperformed the atlas-based contouring software with time saved 1.5 min *vs.* 0.3min. Zhu et al. (59) compared atlas-based with deep CNN-based techniques in aspects of automatic segmentation for multiple OARs, using DSC and MSD as evaluation metrics. In respect of the heart, lungs and liver, there was no significant difference between the atlas-based and the deep CNN-based techniques. As for the spinal cord and the esophagus, the deep CNN-based technique had a superior performance than the atlas-based technique (DSC: 0.71 *vs.* 0.54 & MSD: 2.6 mm *vs.* 3.1 mm for the esophagus; DSC: 0.79 *vs.* 0.71 & MSD: 1.2 mm *vs.* 2.2 mm for the spinal cord). Zhang et al. (64) also compared the CNN-based and atlas-based automatic segmentation techniques. In their study, the CNN-based method performed better than the atlas-based method in the left lung (DSC: 0.948 *vs.* 0.932; MSD: 1.10 mm *vs.* 1.73 mm), heart (DSC: 0.893 *vs.* 0.858; MSD: 1.65 mm *vs.* 3.66 mm) and liver (DSC: 0.937 *vs.* 0.936; MSD: 2.03 mm *vs.* 2.11 mm). The CNN-based method segmented the esophagus with a mean DSC of 0.732 and an average MSD of 1.38 mm, whereas that of the atlas-based method for the esophagus was unavailable.

**Table 9:** Selected works on comparison between atlas-based and deep learning-based automated segmentation.

Reference	Year	Evaluation Metrics	Comparison Results	
			atlas-based	deep learning-based
Lustberg et al. (91)	2017	time saved	7.8 min	10 min
		(compared with manual segmentation)		
Zhu et al. (59)	2018	DSC	heart: 0.90 ± 0.04	heart: 0.91 ± 0.03
			liver: 0.87 ± 0.05	liver: 0.89 ± 0.02
			esophagus: 0.54 ± 0.08	esophagus: 0.71 ± 0.05
			spinal cord: 0.71 ± 0.06	spinal cord: 0.79 ± 0.03
			lungs: 0.95 ± 0.01	lungs: 0.95 ± 0.01
		MSD (mm)	heart: 3.14 ± 1.31	heart: 2.92 ± 1.51
			liver: 3.83 ± 1.74	liver: 3.21 ± 0.93
			esophagus: 2.67 ± 1.26	esophagus: 2.18 ± 0.80
			spinal cord: 3.03 ± 1.57	spinal cord: 1.25 ± 0.23
			lungs: 1.85 ± 0.53	lungs: 1.93 ± 0.51
		95%HD (mm)	heart: 9.53 ± 4.99	heart: 7.98 ± 4.56
			liver: 11.87 ± 5.06	liver: 10.06 ± 4.28
			esophagus: 9.45 ± 4.64	esophagus: 7.83 ± 2.85
			spinal cord: 11.97 ± 6.88	spinal cord: 4.01 ± 2.05
			lungs: 8.07 ± 2.39	lungs: 7.96 ± 2.57
Zhang et al. (64)	2020	average time	2.4 minutes	1.6 minutes
		DSC	left lung: 0.932 ± 0.040	left lung: 0.948 ± 0.013
			right lung: 0.943 ± 0.017	right lung: 0.943 ± 0.015
			heart: 0.858 ± 0.077	heart: 0.893 ± 0.048
			spinal cord: 0.868 ± 0.031	spinal cord: 0.821 ± 0.046
			liver:0.936 ± 0.012	liver: 0.937 ± 0.027
			esophagus: –	esophagus: 0.732 ± 0.069
		MSD (mm)	left lung: 1.73 ± 1.58	left lung: 1.10 ± 0.15
			right lung: 2.17 ± 2.44	right lung: 2.23 ± 2.33
			heart: 3.66 ± 2.44	heart: 1.65 ± 0.48
			spinal cord: 0.66 ± 0.16	spinal cord: 0.87 ± 0.21
			liver: 2.11 ± 1.31	liver: 2.03 ± 1.49
			esophagus: –	esophagus: 1.38 ± 0.44

DSC, Dice similarity coefficient; MSD, mean surface distance; 95%HD, 95% Hausdorff distance.

Considering the comparison results mentioned above, we can summarize that deep learning based automatic segmentation is more accurate and efficient in delineating the multiple OARs in lung cancer. Although it is not yet available to directly apply a deep learning based algorithm to clinical segmentation for OARs in lung cancer RT, we can use the segmentation generated from it as a starting point and manually adjust it to meet clinical guidelines. Overall, deep learning based automatic segmentation can potentially be employed in clinical routines to relieve radiation oncologists of the tedious contouring work.

### CNN, U-Net, and GAN

With the processing of the CNN layers, the level of abstraction of the extracted features gradually increases. Shallower layers grasp local features, while deeper layers capture global features by using convolution kernels whose receptive fields are much broader. Generally speaking, the deeper CNNs can solve more complex issues. However, with the network depth increasing, the degradation phenomenon has been reported. Surprisingly, such degradation phenomenon is not due to overfitting. Besides, continuously adding more layers to a suitable deep network results in bigger training error. Finally, even though the deeper networks perform better, they are difficult to train owing to the gradient vanishing problem. These reveal that not all CNNs are easy to optimize and the network depth is of crucial importance. It is tedious to find the optimal network depth by trial and error. Introducing residual connections to create shortcuts among blocks of layers may be beneficial to achieve fast and stable training. Nevertheless, when the number of layers in CNNs was rather small, the residual connections may not only fail to help reduce the training difficulty, but also decrease the complexity of the network and thus the expressive power. It is worthy of considering whether or not to add residual connections while designing the network architecture.

U-Net is one of the most popular medical image segmentation networks (46). It is possible to train the U-Net to produce precise segmentations with very little labeled training data. Both convolutions and down-sampling operations are usually local operations, meaning that a lot of local operators need to be stacked in a cascade way to aggregate long-range information (92). Meanwhile, the amount of training parameters also increases while stacking them, which becomes a large obstacle to improve the calculation efficiency. Additionally, more down-sampling operations lead to the loss of more spatial information during encoding, resulting in poor accuracy of medical image segmentation. Of course, those issues exist in the decoder as well. In allusion to this instance, some researchers attempt to address those limitations. Wang et al. successfully solved this issue by proposing a novel network architecture called non-local U-Net, which is equipped with flexible global aggregation blocks based on the self-attention operators (92–95).

GAN, as a method of unsupervised deep learning, is commonly used in medical image segmentation. GAN can train any kind of network architecture as its generator network. GAN dispenses with the need for using Markov chain to sample repeatedly, and for inferencing in the learning process, which avoids the difficulty of calculating the probability. But there are some challenges faced by GAN, such as how to break through the non-convergence problem and collapse problem (96) which may occur in the learning process.

### Current Challenges and Limitations

With the significant progress in computer science and techniques, deep learning algorithms play an indispensable role in image segmentation with their compelling ability to extract features automatically. According to studies in recent years, image segmentation technologies based on deep learning have surpassed traditional segmentation methods in segmentation efficiency and accuracy. Nonetheless, deep learning based automatic segmentation techniques still face various challenges and limitations.

#### Low Contrast Issue

Unlike natural images, the information contained in medical images is more complicated, and the similarity between the target and the surrounding background in the image is extremely high for low contrast tissues. Therefore, it is difficult to accurately detect target boundaries or to delineate the target from the background. Besides, to guarantee the RT outcomes, high accuracy is required. So, the first problem to solve is that how to precisely identify the boundaries of low contrast tissues such as esophagus etc. and delineate them with superior performance. Currently, proposed deep learning based automatic segmentation algorithms merely managed to segment high contrast organs in CT with satisfactory results, and usually failed to segment the esophagus with high accuracy. This inferior performance may be explained by the following reasons. First, the appearance of esophagus varies depending on whether it is full of air, of remains of orally given contrast agent, or both (97). Second, the esophagus has certain mobility, which leads to the fact that the esophagus has a greatly inhomogeneous appearance and a versatile shape. Furthermore, studies have indicated that owing to respiration and cardiac motion, esophageal intrafraction motion is generally between 5 and 10 mm and can reach up to 15 mm (98–100). How to improve the inferior segmentation accuracy of the esophagus will become one of the key research directions. MRI has a superior visualization of low contrast tissues compared to CT. Perhaps using MRI data as input could improve the segmentation accuracy to a certain extent. Nevertheless, we ought to put more emphasis on improving existing deep learning segmentation algorithms or coming up with novel ones. Given that 3D-based convolutions could address volumetric homogeneity better and take full advantage of the 3D spatial context compared with 2D-based convolutions, it may be beneficial to use a 3D network to segment the esophagus which has a thin tubular and continuous structure. Fechter et al. (97) have proposed a new scheme and achieved competitive results. Firstly, they employed a 3D-FNN to yield a first estimation of the esophagus. Then, an active contour model and a random walker approach were used to refine the first estimation to the final contour.

#### Size of Dataset

The size of the training dataset greatly affects the robustness of deep learning algorithms. It could be argued that the generalization of deep learning algorithms is expected to increase with an enlarged training dataset. Currently, most studies use different datasets collected individually, except in some segmentation competitions. Besides, most of the datasets reported in this review are not adequate in the era of big data. Taking a limited dataset for training and testing may lead to model over-fitting. Hence the efficient generalization of the proposed algorithms cannot be demonstrated. Utilizing a transfer learning strategy may potentially handle the issue of limited data size. Image-Net is typically employed for pre-training networks to process medical images (101). On the other hand, data augmentation is also an effective method to address the issue of a limited dataset. Furthermore, it is worth considering of establishing a public image dataset with a high-quality ground truth label to make advances in deep learning based automatic segmentation techniques.

However, it is not practical to establish an adequate public image dataset for the initial training of the deep learning model within a short time. At present, the amount of data utilized for the initial training is most likely not adequate. With the accumulation of clinical cases, we can utilize new cases to further fine-tune the deep learning model to achieve better performance. Notably, catastrophic forgetting may occur during the fine-tuning process. While we can solve this issue by retraining the deep learning model utilizing both old and new cases, this approach is tedious and inefficient. Moreover, manual labeling the new cases is also a time-consuming and laborious task. In view of such a situation, Men et al. (102) proposed a novel scheme. In addition to training an automatic segmentation network, they also trained a binary classifier to judge the quality of the automatic segmentation. For a batch of new clinical cases, the segmentation network firstly performs the automatic segmentation. Then, the binary classifier judges the segmentation result and selects the case with a DSC less than the setting threshold. These selected cases are manually labeled by radiotherapy experts and then used to fine-tune the segmentation network to improve its performance. Their scheme remarkably reduces the manual labeling effort and enables the deep learning model to continually update over the accumulation of clinical cases, thus achieving the strategy of continual learning. Their method could be explored to efficiently improve the robustness of deep learning models in the future.

#### Lack of Consensus on Guidelines

Another limitation of deep learning based automatic segmentation technique is that we usually cannot objectively determine whether a clinically acceptable ground truth is an optimal case due to lack of consensus. In general, the shape and position of organs vary greatly among different patients due to race, gender, age and progression of the disease etc. Radiation oncologists manually segment the OARs and GTV to generate the ground truth depending on their own prior knowledge and experience, which leads to inconsistencies in process of generating the ground truth for both inter- and intra-observers. Ground truth plays a vital role in the performance of deep learning algorithms. Moreover, differences in image acquisition protocols (such as posture and breath-hold conditions etc.) could also potentially affect the performance of deep learning algorithms. Besides, it is also necessary to establish international consensus on guidelines to eliminate the inconsistencies that existed in contouring the ground truth for both inter- and intra-observers.

#### Network Design

With the increase of the number of layers used in the network, the deep learning algorithm has stronger feature expressive power, making subsequent predictions easier and more accurate. However, the complexity of the deep learning algorithm also increases simultaneously, which means that the network training must take more time and GPU memory. Furthermore, to extract and integrate multi-scale features, most existing methods attempt to propose and add more complex blocks and strategies to commonly used networks, which significantly increases the GPU memory and computation cost. So, it is worthwhile to think about how to achieve a balance between network design and computation time or cost. In this review, the highest accuracy reported in terms of DSC was achieved by Hu et al. (65) for the lung segmentation. They combined the Mask R-CNN with the K-means kernel to achieve accurate segmentation. With regard to OARs, Han et al. (40) developed a multi-resolution VB-Net architecture and achieved the best performance in segmentation of heart, aorta, trachea, esophagus. Moreover, it is worthwhile to explore to stack networks sequentially to build cascaded architectures or to build multi-level nested architectures. Zhang et al. (103) designed a slice classification model-facilitated 3D encoder-decoder network for segmenting OARs in head and neck cancer. The utilization of the slice classification model alleviates class-imbalance issues existing in small volume OARs, and decreases unnecessary computation time. Qin et al. (104) proposed a two-level nested U-Net structure called U^2^-Net for salient object detection and obtained competitive performance against other state-of-the-art networks at a low GPU memory and computation cost. The researchers who are interested in this domain can grope for novel network architectures so that the performance of segmentation can be improved.

#### Clinical Issues

Deep learning algorithms are referred to as black-box algorithms owing to lack of interpretability. It is hard to fully understand how, and which factors result in poor segmentation performance. Namely, the deep learning algorithm may fail to segment the OARs and GTV in an unpredictable way which is dangerous in clinical practice. Before deep learning based segmentation techniques can be made clinically available, we ought to consider the legal and ethical responsibilities, and the issues of ensuing patient safety. Therefore, it is of vital importance to implement an exhaustive, comprehensive, and rigid quality assurance procedure for deep learning based segmentation techniques to assure adequately high accuracy of the segmentation with complete conformance to a set of safety criteria. Independent scoring software and commercial third-party assessment software may possibly serve as tools to handle the issues originating from automated segmentation algorithms. Lastly, it is also important to guarantee that all generated segmentations information can be exported/imported consistently and accurately to other systems such as the treatment planning system. The limitation of the automatic segmentation techniques should be stated so that the users are aware and vendors can address these issues (105).

## Summary

Deep learning based automatic segmentation techniques have rapidly become the state-of-the-art technique in delineating the OARs and GTV in lung cancer RT. The auto-segmentation of lung, heart and liver has achieved satisfactory results. However, one still has to study how to improve the segmentation performance of esophagus taking into account low contrast and respiration motion and other factors. When it comes to segmentation of the GTV, the studies are rather few thus far and the segmentation performance is poor. We still need to make effort to improve accuracy in delineating the GTV and even the CTV. Deep learning based automatic segmentation is a rapidly developing field. Over the next few years, a further modification of deep learning algorithms may be explored to address the remaining issues and improve the accuracy of segmentation. It holds great promise to employ a deep learning based technique as a highly useful tool to automatically segment the OARs and GTV for routine clinical use under expert visual inspection and approval. The promising result of segmentation potentially contributes to optimizing RT planning and developing adaptive radiotherapy. Finally, cautions must be taken in terms of all aspects of limitations before deep learning based automatic segmentation is used for clinical practice.

## Author Contributions

XL and RY wrote the manuscript. K-WL and L-SG helped with manuscript redaction. RY carried out a technical review of the manuscript in aspects of clinical practice and deep learning, and revised the manuscript. All authors contributed to the article and approved the submitted version.

## Funding

This work is partly supported by the National Natural Science Foundation of China under Grants Nos.11735003, 11975041, and 11961141004, the fundamental Research Funds for the Central Universities, National Key Research and Development Program of China (2021YFE0202500), Capital’s Funds for Health Improvement and Research (2020-2Z-40919), Beijing Municipal Commission of Science and Technology Collaborative Innovation Project (Z201100005620012), and China International Medical Foundation, HDRS2020030206.

## Conflict of Interest

The authors declare that the research was conducted in the absence of any commercial or financial relationships that could be construed as a potential conflict of interest.
